# Cathodoluminescent Analysis of Sapphire Surface Etching Processes in a Medium-Energy Electron Beam

**DOI:** 10.3390/ma15041332

**Published:** 2022-02-11

**Authors:** Arsen Muslimov, Vladimir Kanevsky

**Affiliations:** Shubnikov Institute of Crystallography, Federal Scientific Research Center Crystallography and Photonics, Russian Academy of Sciences, 119333 Moscow, Russia; kanevsky@crys.ras.ru

**Keywords:** patterned sapphire substrate, electron etching, gold, cathodoluminescent analysis, anisotropy, light-emitting diodes, windows

## Abstract

Sapphire crystals are widely used in optics and optoelectronics. In this regard, it is important to study the stability of crystals under external influence and the possibility of modifying their surfaces by external influence. This work presents the results of studying the processes of the action of an electron beam with an average energy of 70 keV or less under vacuum conditions on the surfaces of sapphire substrates of various orientations. The effect of etching a sapphire surface by an electron beam in vacuum at room temperature was discovered. The highest etching rate was observed for A-plane sapphire (the average pit etching rate was 10^−6^ µm^3^/s). It was shown that the rate of etching of a sapphire surface increased many times over when gold is deposited. An in situ method for studying the process of etching a sapphire surface using cathodoluminescence analysis was considered. Possible mechanisms of sapphire etching by a beam of bombarding electrons were considered. The results obtained could be important in solving the problem of the stability of sapphire windows used in various conditions, including outer space. In addition, the proposed method of metal-stimulated etching of a sapphire surface can be widely used in patterned sapphire substrate (PSS) technology and further forming low-dislocation light-emitting structures on them.

## 1. Introduction

Sapphire crystals (corundum, α–Al_2_O_3_), because of the peculiarities of their chemical composition and crystal structure, are able to withstand a range of intense external influences, including high temperature, pressure, and mechanical stress. Sapphire belongs to the trigonal (rhombohedral) crystal system, as a result of which it has a pronounced anisotropy of physical and chemical properties. Sapphire crystals are used mainly in optics and optoelectronics, where the anisotropy of properties is most in demand. For example, for high-strength optical glasses, it is preferable to use C(0001)-plane sapphire with zero birefringence [1]. Furthermore, the C-plane of sapphire has been used for a long time as a substrate for the formation of light-emitting devices [2,3,4]. Thus, using the C-plane sapphire profiling technique, it was possible to increase the light yield of devices by up to 20% [5,6]. However, it was revealed that there is a fundamental problem associated with the use of C-plane sapphire in light-emitting devices, which prevents a further increase in efficiency: the quantum-confined Stark effect [7,8]. The presence of near-surface electric fields in polar structures leads to a loss of efficiency and a long-wavelength shift of the radiation maximum. It is possible to reduce or completely suppress the effect of near-surface electric fields by using semipolar or nonpolar oriented nitride layers. In particular, such structures may be formed on M-plane (10$\bar{1}$0), R-plane (1$\bar{1}$02), and A-plane (11$\bar{2}$0) sapphire [9,10,11,12].

There are several important application requirements for sapphire crystals in general. On the one hand, they are associated with the use of devices operated in a wide variety of environments, including space flight systems, as protective optical glasses. It should be borne in mind that apparatuses are susceptible to various types of ionizing radiation and flows of charged particles of various energies in vacuum conditions. On the other hand, optimization of the pregrowth preparation of the substrate surface (polishing, microstructuring, profiling) is also important in the formation of light-emitting devices. Note that these requirements are related: methods of external action that lead to decomposition or etching of the surface of sapphire plates can be considered as methods of microstructuring and profiling their surface.

A number of works [13,14,15] have been devoted to the study of the processes of influence of ionic flows leading to the transformation of the surfaces of sapphire substrates. The effects of various types of radiation on sapphire optical fibers with a total radiation dose of up to 3.39 mGy has been more widely studied [16]. The effect of electrons is also used in electron lithography for resist processing [17] and in high-energy electron accelerators [18]. In this paper, we present the results of studying the processes of the action of an electron beam with an average energy of 70 keV or less under vacuum conditions on the surfaces of sapphire substrates of various orientations.

Since the processes of surface transformation in the case of external action usually cover the near-surface region of crystals up to tens of microns, contact research methods are not applicable. The only possibility is the use of noncontact methods based on the analysis of electromagnetic radiation from the surface under investigation [19,20]. In particular, the authors of [19] demonstrated the possibility of in situ studies of the processes of transformation of sapphire surface using expensive synchrotron radiation sources. In this paper, we considered the possibility of in situ study of the process of etching the sapphire surface as a result of medium-energy electron bombardment using luminescence analysis methods. This possibility appeared to be due to features of the cathodoluminescence (CL) spectra of sapphire crystals. Anionic defects in sapphire crystals are identified as F- and F^+^-centers, which are oxygen vacancies that have captured two electrons and one electron, respectively, and their complexes. In the ultraviolet (UV) region of the sapphire CL spectrum, bands are observed in the regions of 330–340 nm (F^+^-center) and 410–415 nm (F-center) [21]. Since the F- and F^+^-centers are exclusively intrinsic defects of the crystal structure, their concentration is directly dependent on the structural-phase composition of the crystal. On the contrary, in the long-wavelength part of the sapphire CL emission spectrum, bands associated with Cr and Ti atoms with impurities are traditionally observed, which are less dependent on the structural-phase composition and can appear even in the molten state of Al_2_O_3_. In particular, a narrow line at 694 nm and a broad band peaking at 780 nm are associated with ^2^E–^4^A_2_ and ^2^E–^2^T_2_ transitions in Cr^3+^ and Ti^3+^ [22], respectively. Interestingly, heating or increasing the fluence increases the luminescence intensity in the UV region of the sapphire emission spectrum, while the dependence on electron energy has a maximum [23], after which a decrease is observed. The broadening of the chromium line in the long-wavelength region corresponds to an increase in the temperature of shock-shifted luminescent chromium ions above the stationary temperature of the crystal [24]. A similar broadening is observed for a broad band associated with Ti^3+^ [25]. In general, it is possible to study the processes of sapphire etching using the basics of quantitative luminescent analysis, which assume a linear relationship between the luminescence intensity and the concentration of luminescence centers at low concentrations.

Thus, the independent behavior of color centers of different nature during external action makes it possible to consider cathodoluminescence analysis as a very promising in situ method for studying the processes of rearrangement and etching of the surface of sapphire crystals.

## 2. Materials and Methods

The samples were C-, A-, M-, and R-plane sapphire with one-sided chemical–mechanical polishing. It should be noted that the content of chromium and titanium impurities with a concentration of ~10 ppm found in the sapphire crystal was due to their presence in the initial charge. Two types of wafers were used: (a) initial and (b) after annealing at a temperature of 1400 °C for an hour under atmospheric conditions to recrystallize the surface and reduce the concentration of surface-layer defects introduced during growth, machining, and polishing. Next, a layer of gold about 100 nm thick was formed on an A-plane of sapphire by thermal vacuum deposition (VN-2000 configuration). The sample was then annealed in air Naber tube furnace (Nabertherm, Lilienthal, Germany) for 2 h at 800 °C to form a discrete gold structure.

The study by the method of excitation of CL in the samples was carried out with an electron beam of an EG-75 electron diffraction recorder, the electron energy of which was 40 keV, 50 keV, 60 keV, and 70 keV; the spot diameter of which was 0.5 and 3 mm; the electron flux density of which was 10^21^ cm^−2^∙s^−1^; and the electron beam current of which was 80 μA. This was undertaken at room temperature (special heating of the samples was not performed). The CL spectra of all samples were studied 10 s after the start of irradiation. The vacuum was maintained in the 10^−4^ Pa system. We used AvaSpec-ULS2048x64-USB2 spectrophotometric complex (Avantes). The angle of incidence of the electron beam on the plane of the substrate was 45°, and the angle between the axis of the fiber-optic adapter and the direction of propagation of the incident electron beam was 90°. The time dependence of the CR spectra was studied with the following exposure parameters: numbers of spectra, 21 and 46; time interval between spectra, 8 s.

Microscopic studies of the surface of the samples were carried out on an SEM Leo-1450 scanning electron microscope (Carl Zeiss AG, Oberkochen, Germany) and an Ntegra Aura atomic force microscope (NT-MDT, Zelenograd, Russia) in the modes of semicontact topography and phase contrast, respectively.

## 3. Results and Discussion

### 3.1. Orientation Dependence of the CL Spectra of Sapphire Samples

**Figure 1:**
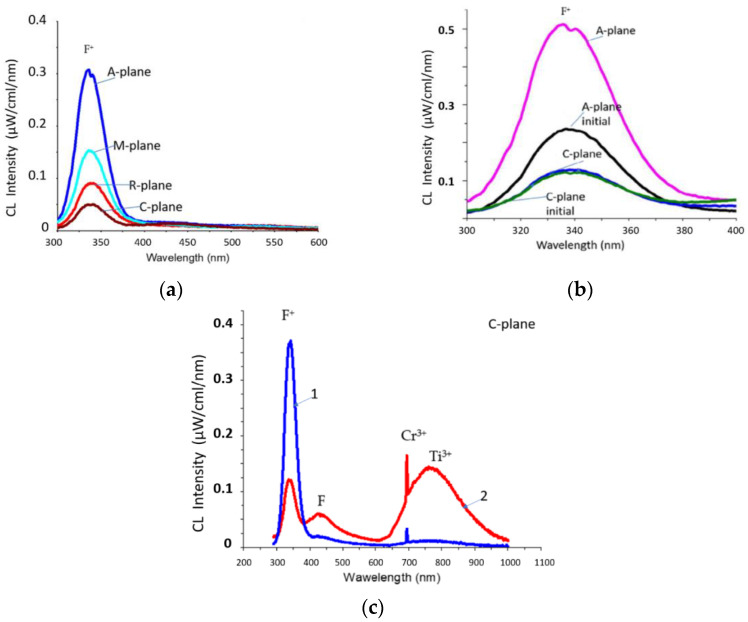
CL spectra of heat-treated at 1400 °C and initial sapphire samples at electron energies of 50 keV (**a**) and 60 keV (**b**). Dependence of the CL spectrum (electron energy 70 keV) of C-plane sapphire on the spot diameter (**c**): 1–0.5 mm; 2–3 mm.

Figure 1a shows the CL spectra (electron energy 50 keV, spot diameter 0.5 mm) of sapphire samples of various orientations after heat treatment at 1400 °C under atmospheric conditions. The spectra showed an intense main F^+^(340 nm)-band, but the F(415 nm)-band was suppressed. In this case, an effect similar to that previously found in [21] was observed. When irradiated with electrons, the F-centers were suppressed because of the formation of new F^+^-centers according to:

F + exposure → (F^+^)^∗^ + e^−^_trap_→ F^+^ + hν (330 nm) + e^−^_trap_ (1)

where (F^+^)^∗^ is an excited F^+^-center.

According to Figure 1a, the absolute intensities I of the luminescence of the CL F^+^-bands were related as follows: I_A-plane_ > I_M-plane_ > I_R-plane_ > I_C-plane_. This corresponds to the ratio of the rates of ion etching of sapphire wafers of different orientations [14] at an accelerating voltage of 30 kV. In both cases, the etching rate was maximum for A-plane sapphire and minimum for C-plane sapphire. Additionally (Figure 1b), the luminescence intensity of the F^+^-band of the CL spectrum was studied for the initial and heat-treated A-plane and C-plane samples (for which the maximum and minimum CL intensities were observed, according to the data in Figure 1a) at a higher electron energy (60 keV). With an increase in energy, electrons were able to penetrate into deeper layers and generate oxygen vacancies in them. Figure 1b demonstrates that the general trend continued: I_A_ > I_C_.

Passing from the luminescence spectra to the processes of electron etching of the sapphire surface, it should be kept in mind that the sapphire substrates were preliminarily annealed to minimize the concentration of oxygen vacancies, and we can assume that the CL intensity in the UV region of the spectrum was proportional to the concentration of oxygen vacancies. In addition, the results showed that an increase in the accelerating voltage led to an increase in the intensity of the emission of F^+^-centers (oxygen vacancies that had captured one electron). In this case, the glow of F-centers (oxygen vacancies that had captured two electrons) was suppressed. Considering that the number of electrons emitted by the cathode every second did not change, the increase in the intensity of the glow of the F^+^-centers could have been associated only with the generation of new oxygen vacancies from deep layers.

The correspondence of the etching processes of a sapphire surface by ion and electron flows, which we found in our experiments, indicated a universal mechanism of defect formation caused by the displacement of atoms during elastic collisions with bombarding particles. In the case of impact mixing, an important factor is the interaction of atoms in the atomic layer. According to accepted calculations [14], the atomic arrangement density is maximum in the C-plane and minimum in the A-plane: 25 and 7.63 at/nm^2^, respectively. The potential energies per atom (the energy of removal of atoms) are 291.23 and 908.63 eV for the A-plane and C-plane, respectively. This explains the maximum rate of ion and electron etching of the A-plane of sapphire. On the contrary, the interplanar distances for the C-plane and A-plane are 2.165 and 1.190 Å, respectively. The binding energy of neighboring atomic layers along any direction in the crystal is inversely proportional to the corresponding interplanar distance. The rate of chemical–mechanical etching depends on the interplanar distance, and since this distance is maximum along C[0001], the etching rate is maximum. The hardness is minimal for C-plane sapphire.

Analysis of the CL spectra of the original and heat-treated samples (Figure 1b) showed a strong difference for A-plane sapphire but similarity for C-plane sapphire. According to [26], after chemical–mechanical polishing, the A-plane and R-plane of sapphire have the highest roughness, and the C-plane of sapphire, the lowest. The low intensity of the F^+^-band for the original A-plane sapphire is associated with a high surface roughness. A significant part of the bombarding electrons is scattered by surface inhomogeneities without the formation of oxygen vacancies. After heat treatment, the surface becomes atomically smooth, the scattering of electrons by inhomogeneities decreases, and the rate of generation of oxygen vacancies increases. On heat-treated A-plane sapphire (Figure 1), there was a bifurcation of the F^+^-peak, which was associated with the presence of luminescence components along and perpendicular to the main optical C-axis of the sapphire crystal. On the initial samples, the splitting of the F^+^-peak was also not observed because of the strong scattering of electrons by surface defects. On the contrary, there was a significant similarity between the CL spectra of the original and heat-treated C-plane sapphire (Figure 1b). The basic (C-plane) sapphire is characterized by close-packed oxygen layers, and it is these layers that determine the features of the corundum-type crystal structure.

An interesting result was obtained in studying the dependence of the spectral features of CL on the diameter of the electron spot on sapphire samples. Figure 1c shows a typical picture of the evolution of the CL spectrum as the electron spot expanded from 0.5 mm to 3 mm. Bands in the UV region underwent radical changes: the F^+^-band undergoes a reverse transition to the F-band according to the equation F^+^ +e^−^ → F. It is important to note in Figure 1c a weak Ti^4+^ peak (435 nm, curve-2) next to the F-peak on the right, which is detected when the spot expands to 3 mm. This peak, according to [27], is associated with F centers in sapphire: radiation stimulated capture of an electron by the F^+^-center with Ti^3+^ to form the F-Ti^4+^ complex. In addition, the intensity of the bands in the long-wavelength region increases sharply (transitions in Cr^3+^ and Ti^3+^). Moreover, the increase in the intensity of the Cr^3+^ and Ti^3+^ bands was proportional to the increase in the spot diameter. It can be assumed that upon defocusing (an increase in the diameter of the electron beam), saturation of the active Ti^3+^ and Cr^3+^ ions was not achieved, and the number of emitting centers was proportional to the cross-sectional area of the beam of electrons that excited luminescence.

**Figure 2:**
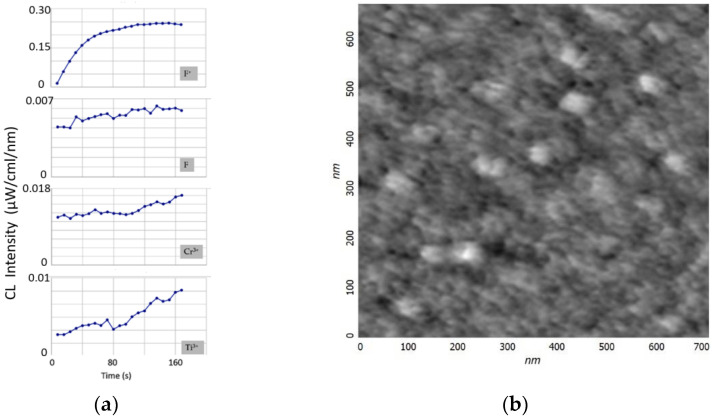
Time dependence (**a**) of the main bands (F^+^, F, Cr^3+^, and Ti^3+^) in the CL spectra for A-plane sapphire (electron energy 40 keV). AFM image (**b**) of a sapphire surface after processing in an electron beam.

To analyze the etching processes of the sapphire surface, the time dependence of the CL spectra was studied (Figure 2). The electron energy was assumed to be as low as 40 keV to reduce the electron beam instability error. The spot diameter was 0.5 mm. The intensity of the F^+^-band, which was proportional to the concentration of oxygen vacancies, reached saturation level after the initial amplification. This was due to a set of processes occurring on the sapphire surface during irradiation, which were determined by the cross-sections of electron scattering, ionization, capture, and excitation, as well as the recombination and clustering of defects [23]. For the rest of the bands, a slight increase was observed over the entire interval of the study. The amplification of the bands could be associated with the process of partial etching of the sapphire surface and an increase in the number of active unsaturated luminescence centers. Thus, in the process of irradiation, an effect similar to the defocusing (diameter increase) of the electron spot was observed. The increase in the surface area was small, and therefore, the enhancement of the F, Cr^3+^, and Ti^3+^ bands was rather weak. Moreover, it was not possible to detect the etching area by microscopic methods. Figure 1b shows an AFM image of an A-plane sapphire surface after processing in an electron beam, but it is difficult to judge the process of its modification. The surface roughness of sapphire after electron bombardment was less than 0.2 nm. This corresponded to the surface roughness of the initially atomically smooth sapphire substrates. It turned out to be impossible to obtain a high-quality image using electron microscopy (SEM) because of the charging of the dielectric surface of the sapphire.

It is possible, by analogy with [28], that the processes of sapphire etching can be visualized only under long-term electron irradiation (energy 100 keV) for more than 30 min with parallel heating of the sample to at least 1023 K, which is a technologically difficult task.

In the work presented here, it was proposed to accelerate the process of etching sapphire in a beam of bombarding electrons using metal stimulators, particularly gold nanocrystals. The choice of gold was due to the following factors. Gold has weak adhesion to sapphire and, when heated, forms a discrete structure of nano- and microcrystals [29]. The successful implementation of the etching process with metal stimulators could approach a cheaper technology for profiling sapphire. In addition, according to the phase diagram [30], there is a wide range of solid solutions in the gold-rich part of the Al–Au system. There are low-melting phases, such as the rhombohedral phase of Al_2_Au_5_; an intermediate- to high-temperature disordered β-phase of the bcc-type, which, upon cooling to 400 °C, transforms into a low-temperature α-phase of AlAu4; a distorted βMn-type phase AlAu_4_ with a solubility ranging from 80 to 81.2 at.% Au; and an Au fcc-type solid solution with a maximum solid state solubility of 16 at.% Al at 545 °C.

In the case of oxygen desorption, the outflow of positively charged aluminum atoms to negatively charged gold crystals can accelerate the decomposition (etching) of the sapphire surface. In addition, there have been recent studies [31] in which gold nanocrystals were used to obtain unstrained GaN films on sapphire.

### 3.2. A-Plane Etching of Sapphire with Gold Nanocrystals in an Electron Beam with an Energy of 40 keV

**Figure 3:**
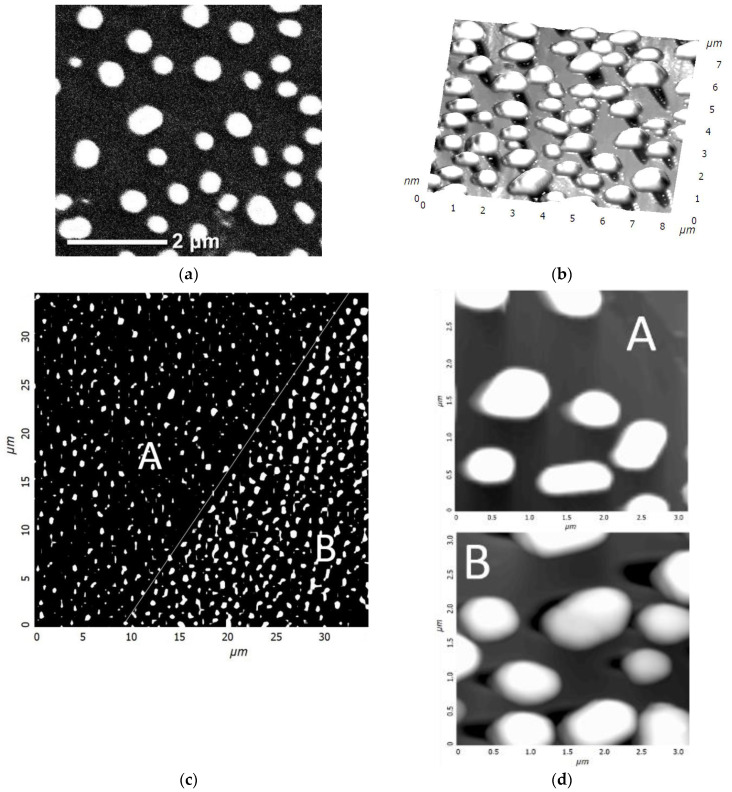
SEM image of A-plane sapphire with gold nanocrystals (**a**). AFM image (3D) of the A-plane of a sapphire with gold nanocrystals after exposure to electrons (**b**). AFM images of adjacent areas A (not treated with electrons) and B (treated with electrons): 35 × 35 µm^2^ (**c**), 3 × 3 µm^2^ (**d**). The thin white line marks the border of regions A and B.

**Figure 4:**
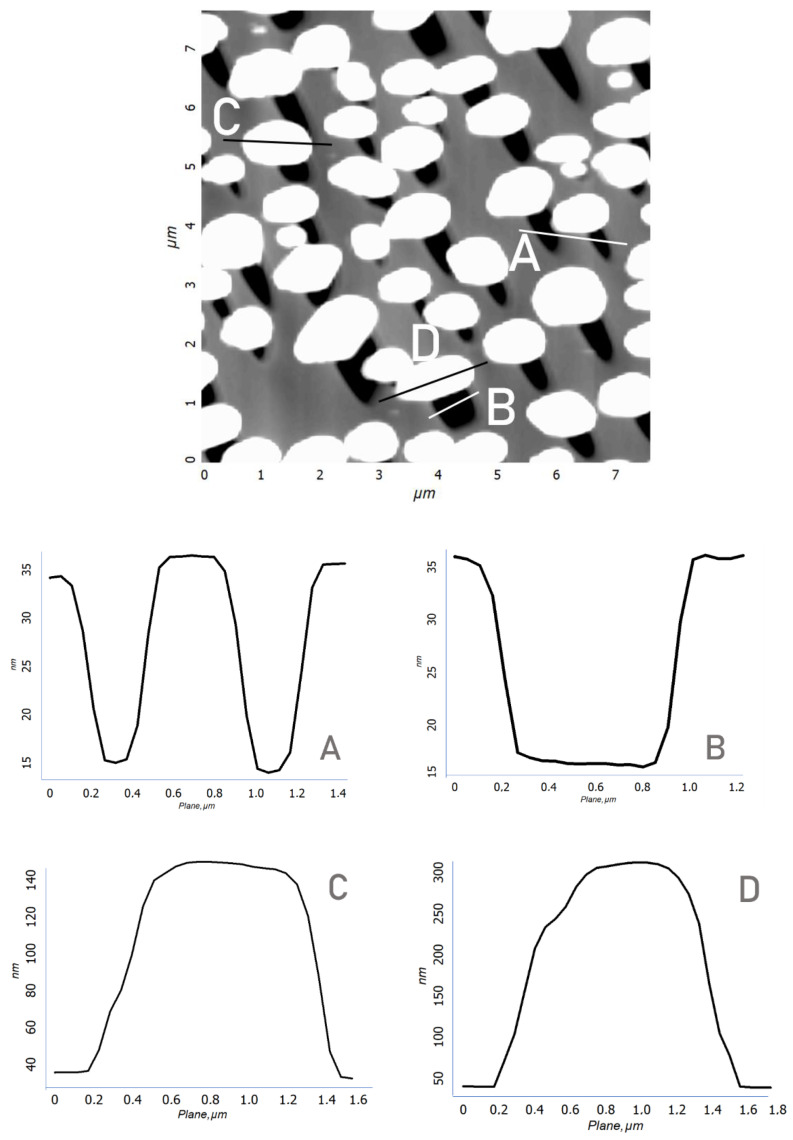
AFM image of an A-plane sapphire with gold nanocrystals after exposure to electrons. Topographic sections of etch pits (**A**,**B**) and of an individual Au nanocrystal (**C**,**D**).

In the process of depositing a gold film on A-plane sapphire and heat-treating it, a discrete structure of nanocrystals with lateral sizes from 300 to 700 nm and heights of up to 250 nm was formed (Figure 3a). At the next stage, the sample surface was irradiated with a focused electron beam with an accelerating voltage of 40 kV (beam diameter 0.5 mm) without special heating for 400 s. When examining the surface of the irradiated sample by scanning electron microscopy (SEM), no significant changes were found. Only a slight rounding of gold nanocrystals was observed. A more detailed picture of the effect of electron flows was visualized by scanning using atomic force microscopy (AFM). Etch pits were found in the region of the sapphire surface adjacent to the gold nanocrystals (Figure 3b). The etch pits had an elongated, almost triangular shape. In addition, there were sharp morphological differences (Figure 3c,d) between the areas of treated and untreated electrons. First of all, an increase in the size of nanocrystals was noticeable. A distinctive feature of the etch pits (Figure 4) was that even with different linear dimensions of the pits, they were of the same depth of about 10 nm. This fact indicates the mechanism of layer-by-layer removal of material through intermediate cracking processes [32]. Cracking is caused by local displacement of aluminum atoms and desorption of oxygen atoms, which weaken the interatomic bonds in the lattice. According to our AFM data, the average etching rate of an individual pit was calculated in the order of 10^−6^ µm^3^/s.

**Figure 5:**
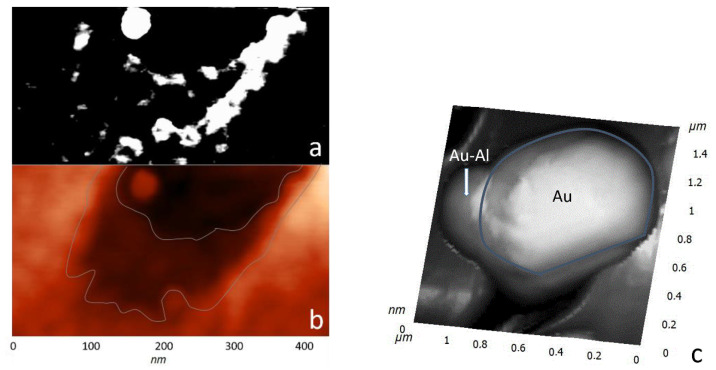
AFM images of an individual etch pit: (**a**)—phase contrast mode; (**b**)—topography mode; (**c**)—3D image of a gold nanocrystal on sapphire after electron impact.

Interesting results were obtained during the study by probe microscopy in the phase contrast mode, which made it possible to contrastingly display materials of various natures. In Figure 5, one can see the accumulation of material along the borders of a pit with two bases. The high contrast indicates significant differences in the mechanical properties of the cluster materials and the A-plane sapphire surface. Given that the saturated vapor pressure of aluminum is low, oxygen was desorbed during the dissociation of the sapphire surface, and aluminum formed clusters. The accumulations of aluminum had a loose structure, which is the reason for the high contrast of the phase picture. The bond between the aluminum atoms and the sapphire surface was maximum at the boundaries of an etch pit; therefore, aluminum accumulations decorated the profile of the pit.

The reduced aluminum atoms were positively charged and diffused to the negatively charged gold islands. The negative charge flowing down from the sapphire surface to the gold islands reduced the effect on the incident electron flux and contributed to the continuity of the etching process. The flow of aluminum atoms to the gold nanocrystal was associated with an increase in their size in the base region (Figure 5b). This was also the reason for the observed rounding of gold nanocrystals when examined by SEM. In the area of the base, a precipitate was formed by compounds of the Al–Au system. According to the phase diagram [30], the most probable compound in the gold-rich region is AlAu_4_. However, intense diffusion of the Al and Au components of the solid solution would require heating of this region to ~400–500 °C. It is known that in thin layers, all processes proceed at significantly low temperatures. A refined diagram [33] demonstrates that, in nanometer layers, the phase composition in the gold-rich part suggested several Al_2_Au_5_ and AlAu_4_ compounds, and the temperature of active diffusion processes was minimized to 100–200 °C. Considering that the reactions of formation of intermetallic compounds in the Au–Al system are exothermic [33] (they proceed with the release of heat), the Au–Al deposit formation mechanism can be self-sustaining.

**Figure 6:**
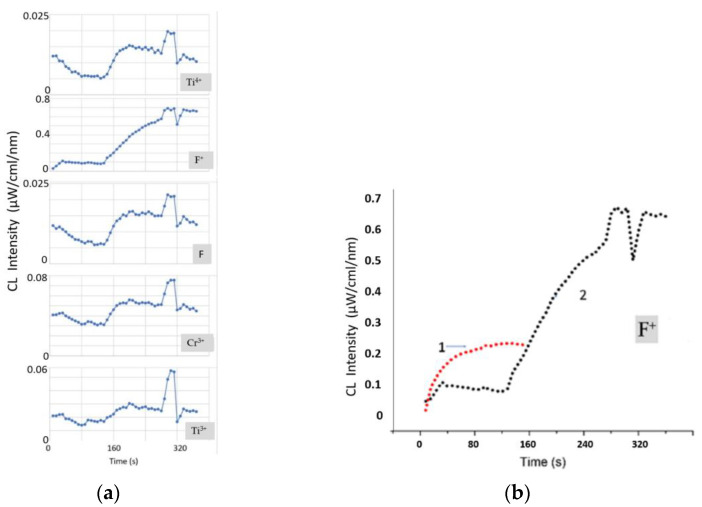
Time dependence of the main bands (Ti^4+^, F^+^, F, Cr^3+^, Ti^3+^) in the CL spectra for A-plane sapphire (electron energy 40 keV) with gold nanocrystals (**a**). Comparison (**b**) of the time dependences of the F^+^-bands in the CL spectra (electron energy 40 keV) for pure A-plane sapphire (curve 1) and that with gold nanocrystals (curve 2).

The time dependence of the CL spectra (Figure 6) of an A-plane sapphire sample with gold nanocrystals was studied. For a correct comparison with a pure A-plane sapphire sample without gold, the same beam parameters were used—electron energy of 40 keV and a spot diameter of 0.5 mm. The intensity of the F^+^-band of CL, which was proportional to the concentration of oxygen vacancies at the initial stage, also reached the saturation level. However, its absolute value was much lower than in the sample without gold. Gold covered a significant part of the A-plane surface, and active desorption of oxygen proceeded only from the uncovered part. The flow of electrons to gold nanocrystals could also have played an important role. The charge flowed to the gold nanocrystals until their potential was equal to the potential of the cathode. Calculations taking into account the condition of equality of potentials, the electrical capacitance of gold nanocrystals, and the beam parameters showed that their full charging occurred in fractions of a second. This allows us to state that the process of oxygen desorption began from the first seconds, but until the moment (130 s—Figure 6a), it did not have a large-scale character and did not lead to an increase in surface roughness. The intensity of the Cr^3+^ and Ti^3+^ bands at the initial stage did not change and even partially decreased. From 130 s, etching began with the formation of sapphire pits, and the intensity of the Cr^3+^ and Ti^3+^ bands increased. It is interesting that an increase in the etched surface of sapphire was observed up to 200 s, after which the F, Cr^3+^, and Ti^3+^ bands reached the saturation level, and the rate of increase in the intensity of the F^+^-band decreased (Figure 6b). The rate of generation of oxygen vacancies at this stage increased to such an extent that the reverse recombination of vacancies became an important factor. A focused electron beam transferred all active Cr^3+^ and Ti^3+^ ions located in the illumination region into an excited state and, as a result, saturation of the bands was observed.

Of particular interest is the behavior of the F and Ti^4+^ bands (Figure 6a). The positions of their features on the time dependence coincided with the positions of the other bands, but there were also differences. Note that the F and Ti^4+^ bands indeed luminesced in the complex (their curves were identical). Preliminary annealing of samples in air led to the oxidation of Ti^3+^ centers concentrated in the surface layer to Ti^4+^. Therefore, the concentration of Ti^4+^ centers dominated at the initial stage. During irradiation, the Ti^4+^ + e^−^ → (Ti^3+^)*→ Ti^3+^ + (730 nm) transition took place. This was associated with a decrease in the intensity of the Ti^4+^ band at the initial stage. In addition, the transfer of electrons to F^+^-centers from Ti^4+^ centers with the formation of F-centers seemed to be more feasible in sapphire than a direct transition due to electron bombardment. Therefore, a decrease in the intensity of the F–Ti^4+^ complex is observed.

The burst of intensity in all CL bands at 280 s remains incomprehensible (Figure 6). The sharpest surge was observed for the Cr^3+^ and Ti^3+^ bands. Analysis of these bands also demonstrated their maximum broadening at this stage, which indicated the maximum local temperature. It can be assumed that only nearby aluminum atoms, which formed in the etch pit after dissociation of the sapphire surface, diffused to the gold nanocrystal. The most distant ones formed positively charged clusters. Over time, as the clusters grew, their repulsion from each other and from the boundaries of the etch pit, where aluminum accumulations were also observed, increased. At the same time, the force of attraction of aluminum clusters to negatively charged gold nanocrystals increased. As time passed, distant aluminum clusters massively diffused to the gold nanocrystals. Because of multiple reactions of the formation of the compounds Al_2_Au_5_ and AlAu_4_, sharp heating was observed in the region of the gold nanocrystal. This contributed to a surge in the intensity of all bands at 280 s and their simultaneous attenuation upon reaching 320 s. It is also important to note a multiple increase in the intensity of CL in the UV region of the spectrum when an A-plane sapphire sample was coated with gold nanocrystals in comparison with a pure A-plane sapphire sample (Figure 6b). This result confirms the importance of the factor of additional heating of the surface due to reactions in the Au–Al system.

**Figure 7:**
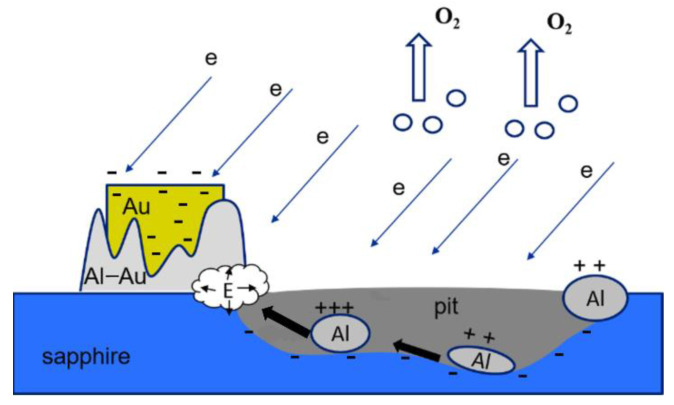
Scheme of A-plane etching of sapphire with gold nanocrystals in the process of exposure to electrons.

In general, the scheme of A-plane etching of sapphire with gold nanocrystals in the process of exposure to electrons can be represented as follows (Figure 7):

### 3.3. Effects of Inelastic Scattering of Electrons on the Surface of Sapphire

In explaining the results obtained, we took as a basis the mechanism of defect formation caused by the displacement of atoms during elastic collisions with bombarding electrons. Subsequently, because of the weakening of interatomic bonds in the lattice, layer-by-layer removal of material occurs through intermediate cracking processes within the atomic plane. The question arose: are the bombarding electrons capable of leading to displacement of atoms in the sapphire lattice? Calculations [34] showed that the energies required to displace aluminum and oxygen atoms from the sapphire lattice were 18 and 75 eV, respectively. It is possible to consider the kinetic mechanism of electron-stimulated desorption of atoms within which the kinetic energy E_k_ transferred to an atom of a solid upon collision with an incident electron is determined by the equation:

(2) $$E_{k}=E_{0}\frac{4m_{e}m_{a}}{{\left(m_{e}+m_{a}\right)}^{2}}\cos^{2}\left(\varphi \right)$$

where m_a_ and m_e_ are the masses of the atom and electron, respectively; E_0_ is the energy of the incident electron; and φ is the scattering angle. An estimate based on the collision theory shows that when the sapphire surface is bombarded by 40 keV electrons incident at an angle of 45°, the kinetic energies E_k_ transferred to oxygen and aluminum atoms are ~2.9 eV and 1.7 eV, respectively. Thus, elastically scattered electrons cannot cause the observed etching processes. The thermally activated movement (displacement) of atoms caused by the heating of the sapphire surface under the action of an electron beam can also be considered. Estimates given in [35,36] indicate that the heating of a sapphire surface under the influence of a bombarding electron flow reaches no more than a hundred degrees Celsius. Such heating could have contributed to the acceleration of exothermic reactions in the Al–Au system, but it would have been of negligible effect for the decomposition of the sapphire surface. Presumably, in our case, the effects of inelastic electron scattering were observed. The most probable process is the radiolytic decomposition of sapphire, which is based on the Auger decay effect [37]. More specifically, the process of radiolysis can be represented as follows: as a result of the impact of an external electron, a hole is formed in the inner electron shell Al(2p) of the aluminum ion. After that, one valence electron in O(2p) of the O^2−^ anion jumps into this hole with the release of one or two additional anionic oxygen valence electrons. As a result, the O^2−^ anion changes its charge state and is displaced from the Al_2_O_3_ lattice. Comparing our data (electron energy 40 keV) with the results of [28], where etching was observed at a higher energy of 100 keV and a duration of 30 min, it can be noted that the cross-section of inelastic electron scattering in a dielectric medium, which causes its radiolysis, decreases with increasing electron energy [38,39]. It can be argued that a decrease in the energy of electrons at energies below 100 keV enhances the processes of radiolysis of the sapphire surface. Heating above 1000 K used in [28] promotes only intense desorption of oxygen and does not directly affect the radiolysis process.

## 4. Conclusions

Sapphire crystals are widely used in optics and optoelectronics. When using them as optical glasses of devices operated in a wide variety of environments, including spacecraft, resistance to the effects of various types of ionizing radiation in vacuum conditions and flows of charged particles of various energies is required. On the other hand, technological optimization of the stages of pregrowth preparation of the surface of sapphire substrates (polishing, microstructuring, profiling) that are important in the formation of light-emitting devices is required. In the present work, the results of studying the processes of the action of an electron flow under vacuum conditions on the surface of sapphire substrates of various orientations are presented. The effect of etching a sapphire surface at room temperature in a vacuum by an electron beam with an energy of 70 keV or less was discovered. It was shown that the highest etching rate was observed for the A-plane of sapphire, while the lowest was observed for the C-plane of sapphire. A technique for metal-stimulated etching of a sapphire surface is proposed in which the etching rate increased many times over. The stimulation process was based on exothermic reactions of formation of intermetallic compounds with aluminum reduction on the sapphire surface. For in situ study of the process of etching a sapphire surface, the technique of cathodoluminescence analysis was used for the first time. Possible mechanisms of sapphire etching by a beam of bombarding electrons were considered. An estimate based on the collision theory showed that the bombardment of the sapphire surface by electrons with an energy of 70 keV or less could not have caused the observed etching processes. A sapphire surface etching mechanism based on the effects of inelastic electron scattering is proposed. The most probable process was the process of radiolytic decomposition of sapphire (Auger decay effect). The results obtained in this work could be important in solving the problem of resistance to external radiation effects of sapphire windows of spacecraft. In addition, the proposed method of metal-stimulated etching of a sapphire surface could be widely used in PSS technology and further forming low-dislocation light-emitting structures on patterned sapphire substrate.

## Data Availability

Not applicable.
